# Association of a common genetic variant in RNASEL and prostate cancer susceptibility

**DOI:** 10.18632/oncotarget.20646

**Published:** 2017-09-05

**Authors:** Li Zuo, Ke-Wei Ren, Yu Bai, Li-Feng Zhang, Jian-Gang Zou, Xi-Hu Qin, Yuan-Yuan Mi, Atsushi Okada, Takahiro Yasui

**Affiliations:** ^1^ Department of Urology, Changzhou No. 2 People’s Hospital Affiliated to Nanjing Medical University, Changzhou 213003, China; ^2^ Department of General Surgery, Changzhou No. 2 People’s Hospital Affiliated to Nanjing Medical University, Changzhou 213003, China; ^3^ Department of Urology, Third Affiliated Hospital of Nantong University, Wuxi 214041, China; ^4^ Department of Nephrourology, Nagoya City University Graduate School of Medical Sciences, Aichi 4678601, Japan; ^5^ Department of Orthopedics, The Affiliated Jiangyin Hospital of Southeast University Medical School, Jiangyin 214400, China

**Keywords:** RNASEL, polymorphism, prostate cancer, meta-analysis

## Abstract

The RNASEL gene (2’, 5’-oligoisoadenylate synthetase-dependent) encodes a ribonuclease that plays a significant role in the apoptotic and antiviral activities of interferons. Various studies have used polymorphisms in the RNASEL gene to evaluate prostate cancer risk but studies that show an association between RNASEL Arg462Gln (1385G>A, R462Q, rs486907) polymorphism and prostate cancer risk are somewhat inconclusive. To assess the impact of RNASEL Arg462Gln polymorphism on prostate cancer risk, we conducted a meta-analysis of all available studies including 11,522 patients and 10,976 control subjects. The overall results indicated no positive association between the variant and prostate cancer risk. However, in a subgroup analysis by ethnicity, obvious associations were observed in Hispanic Caucasians for allelic contrast (OR = 1.18, 95% CI = 1.00 - 1.39, *P*_heterogeneity_ = 0.010), homozygote comparison (OR = 1.50, 95% CI = 1.02 – 2.20, *P*_heterogeneity_ = 0.001), and the recessive genetic model (OR = 1.44, 95% CI = 1.01 - 2.05, *P*_heterogeneity_ = 0.002) ; and in African descendants for homozygote comparison (OR = 2.59, 95% CI = 1.29 – 5.19, *P*_heterogeneity_ = 0.194) and the recessive genetic model (OR = 2.61, 95% CI = 1.30 – 5.23, *P*_heterogeneity_ = 0.195). In conclusion, the RNASEL Arg462Gln polymorphism may contribute to the risk of developing prostate cancer in African descendants and Hispanic Caucasians. Further larger and well-designed studies are warranted to evaluate this association in detail.

## INTRODUCTION

Prostate cancer (PCa) is one of the most common types of neoplasm in the Western world. In United States, prostate cancer is the most prevalent cancer, with 217,730 new cases predicted to occur in 2010 [1]. The etiology of PCa is still poorly understood, but exposure to hormones, infectious agents, or dietary carcinogens may contribute to inflammation of the prostate [2, 3]. Intraprostatic inflammation may affect the tissue microenvironment, promoting genetic damage, and driving cellular proliferation, which may lead to prostate carcinogenesis [4, 5]. Prior studies have suggested that family history is the most reproducible and significant risk factor. Men with a brother or father diagnosed with PCa were twice as likely to develop this cancer as men with no relatives affected [6].

Ribonuclease L (RNASEL), which is considered to be a tumor-suppressor gene, plays a significant role in the pathogenesis of prostate cancer through inflammation and infection. RNASEL is on chromosome 1q24-25 and encodes Ribonuclease L, a significant enzyme of the interferon-induced antiviral 2-5A system [7]. Mutation of RNASEL can lead to dysfunction of Ribonuclease L in regulating single-stranded RNA cleavage, cellular viral defense, and tumor suppressor activities, such as stress-mediated apoptosis and regulation of protein synthesis [8–9].

Extensive epidemiological studies had been conducted to explore the association between RNASEL polymorphism and prostate cancer risk. A G-to-A transversion at nucleotide position 1385 (rs486907), which results in a glutamine instead of arginine at amino acid position 462 (R462Q), is one of the most widely investigated polymorphisms in RNASEL. Nevertheless, the association between the RNASEL R462Q polymorphism and prostate cancer risk is controversial because of conflicting case–control studies. Therefore, in this meta-analysis from all eligible studies published to date [10–32], we used enhance statistical power to understand the effect of this variant.

## RESULTS

### Study characteristics

**Figure 1:**
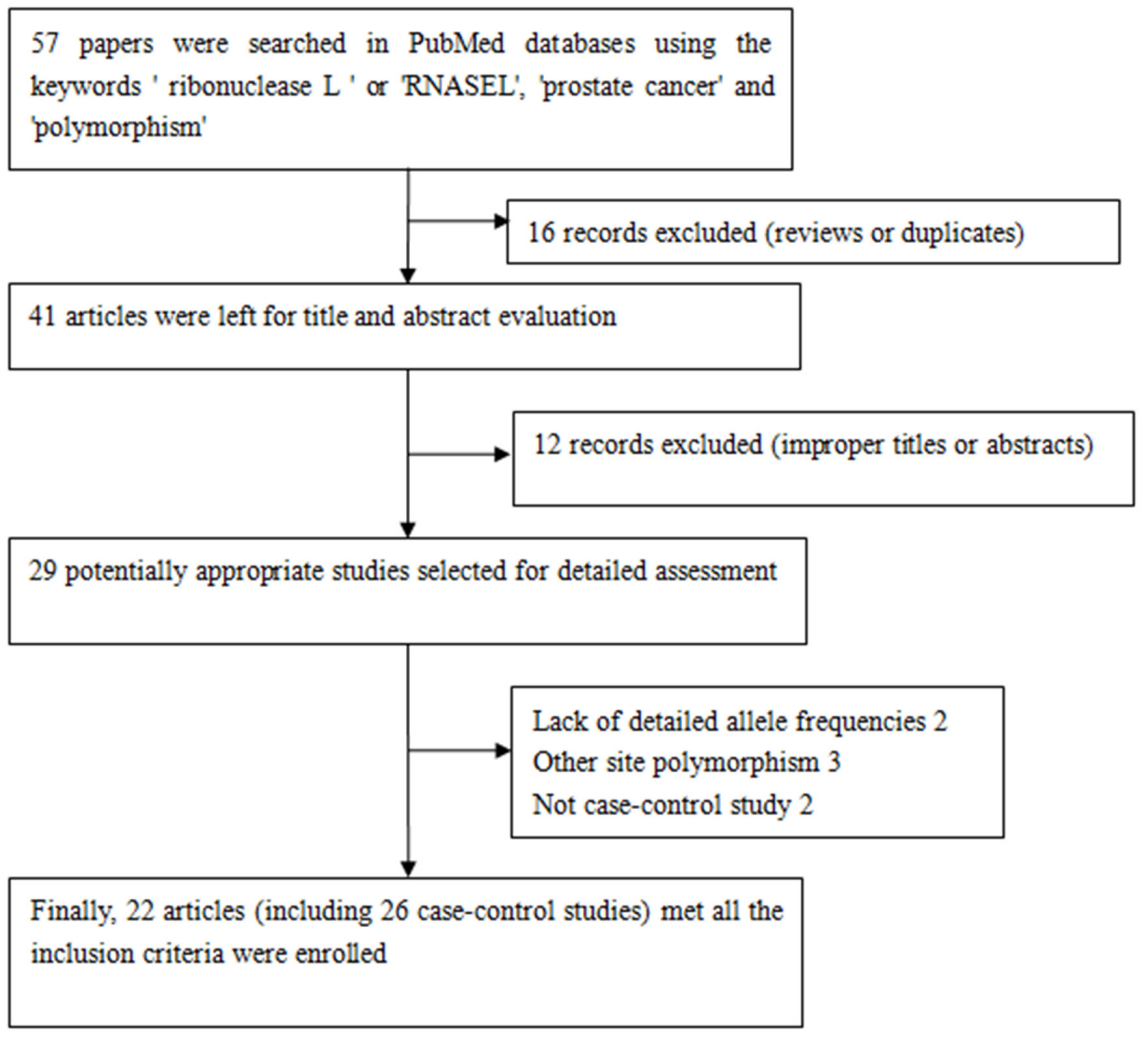
Flowchart illustrating the search strategy used to identify association studies for RNASEL Arg462Gln polymorphism and prostate cancer risk

**Figure 2:**
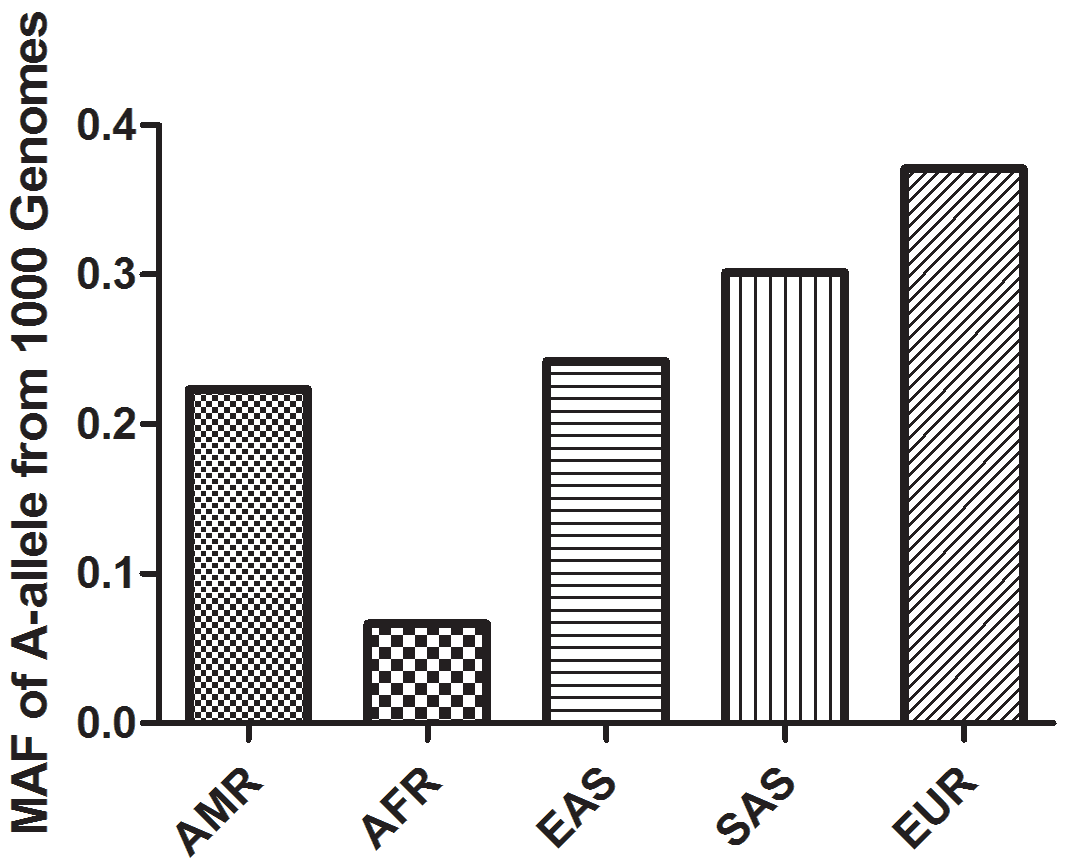
A-allele frequencies for the RNASEL Arg462Gln polymorphism in the controls stratified by ethnicity Vertical line, A-allele frequency; Horizontal line, ethnicity type.

A total of 22 articles (including 26 case–control studies) met all the inclusion criteria and were included (Figure 1). The genotype distribution of the control population was consistent with Hardy-Weinberg equilibrium (HWE) in 19 of the publications. Characteristics of the eligible studies are summarized in Table 1. In general, 11,522 prostate cancer patients and 10,976 control subjects with the RNASEL Arg462Gln polymorphism were evaluated. In the ethnic subgroups, 17 case–control studies were performed with European descendants, three with Asian descendants, and four with African descendants. We checked the Minor Allele Frequency (MAF) reported for the five main worldwide populations in the 1000 Genomes Browser: East Asian, 0.2421; European, 0.3708; African, 0.0666; American, 0.2233; and South Asian, 0.3016. The MAF in our analysis was 0.3034 and 0.2900 in the case and control group, respectively (Figure 2). Hospital-based controls were carried out in 15 of the studies. TaqMan real-time polymerase chain reaction (PCR), the classical genotyping method, was utilized in 10 comparisons. Five studies used the GoldenGate platform or Sequenom MassARRAY platform genotyping method. Six publications had genotype frequency information for familial and sporadic prostate cancer cases.

**Table 1:** Study characteristics of RNASEL Arg462Gln (1385G>A) polymorphism included in this meta-analysis

First author	Year	Country	Ethnicity	Source of	Genotype method	Sample size of case				Sample size of control							
				control		GG	GA	AA	Total	MAF	HWE	GG	GA	AA	Total	MAF	HWE
Babaei	2015	Iran	Asian	HB	PCR	20	15	5	40	0.313	0.421	44	32	4	80	0.250	0.551
Alvarez-Cubero	2015	Spain	Hispanic	HB	Goldengate assay	80	120	37	237	0.409	0.468	61	114	41	216	0.454	0.342
Winchester	2015	USA	Non-Hispanic	PB	Goldengate assay	352	407	105	864	0.357	0.445	330	372	129	831	0.379	0.157
San Francisco	2014	Chile	Hispanic	PB	Taqman	43	31	9	83	0.295	0.351	28	14	4	46	0.239	0.267
Reza	2012	Iran	Asian	HB	Taqman	64	73	44	181	0.445	0.014	14	4	1	19	0.158	0.364
Sakuma	2011	USA	Caucasian	HB	Real-time PCR	43	55	12	110	0.359	0.366	11	21	8	40	0.463	0.723
Beuten	2010	USA	Hispanic	HB	Goldengate assay	75	64	17	156	0.314	0.550	126	91	7	224	0.234	0.048
Meyer	2010	USA	Caucasian	PB	Sequenom MassARRAY	529	547	159	1235	0.350	0.346	505	546	159	1210	0.357	0.551
Martinez-Fierro	2010	Mexico	Mixed	HB	Taqman	9	2	0	11	0.091	0.041	8	2	1	11	0.182	0.197
Agalliu	2010	USA	Non-Hispanic	PB	Pyrosequencing	467	414	84	965	0.302	0.566	572	556	109	1237	0.313	0.110
Wang	2009	USA	Caucasian	PB	Taqman	100	121	27	248	0.353	0.282	88	132	33	253	0.391	0.130
Fischer	2008	Germany	Non-Hispanic	HB	Real time PCR	51	29	7	87	0.247	0.331	42	24	4	70	0.229	0.816
Robbins	2008	USA	African	HB	Sequenom MassARRAY	183	55	5	243	0.134	0.718	225	66	5	296	0.128	0.950
Shea	2008	USA	African	PB	PCR	187	41	2	230	0.098	0.881	362	88	2	452	0.102	0.168
Shook	2007	USA	African	HB	Taqman	45	13	10	68	0.243	<0.001	111	31	3	145	0.128	0.633
Shook	2007	USA	Hispanic	HB	Taqman	72	62	16	150	0.313	0.629	136	96	7	239	0.230	0.039
Shook	2007	USA	Non-Hispanic	HB	Taqman	187	183	60	430	0.352	0.162	221	225	57	503	0.337	0.981
Cybulski	2007	Poland	Non-Hispanic	PB	PCR-RFLP	245	376	116	737	0.412	0.153	177	252	82	511	0.407	0.625
Daugherty	2007	USA	Non-Hispanic	HB	TaqMan	463	505	148	1116	0.359	0.578	554	602	188	1344	0.364	0.235
Daugherty	2007	USA	African	HB	TaqMan	73	23	2	98	0.138	0.905	277	98	5	380	0.142	0.261
Maier	2005	Germany	Non-Hispanic	PB	PCR	133	171	59	363	0.398	0.746	73	97	37	207	0.413	0.629
Nam	2005	Canada	Mixed	PB	Mass spectrometry	477	409	110	996	0.316	0.117	521	459	112	1092	0.313	0.464
Wiklund	2004	Sweden	Non-Hispanic	PB	TaqMan	597	778	247	1622	0.392	0.804	297	384	115	796	0.386	0.611
Nakazato	2003	Japan	Asian	HB	PCR	69	32	0	101	0.158	0.059	71	26	8	105	0.200	0.020
Rokman	2002	Finland	Non-Hispanic	HB	PCR	88	106	39	233	0.395	0.464	69	84	23	176	0.369	0.745
Wang	2002	USA	Caucasian	PB	PCR	389	427	102	918	0.344	0.347	193	233	67	493	0.372	0.802

HWE: Hardy-Weinberg equilibrium of controls, HB: Hospital-based; PB: Population-based; MAF: Minor Allele Frequency.

### Quantitative synthesis

**Figure 3:**
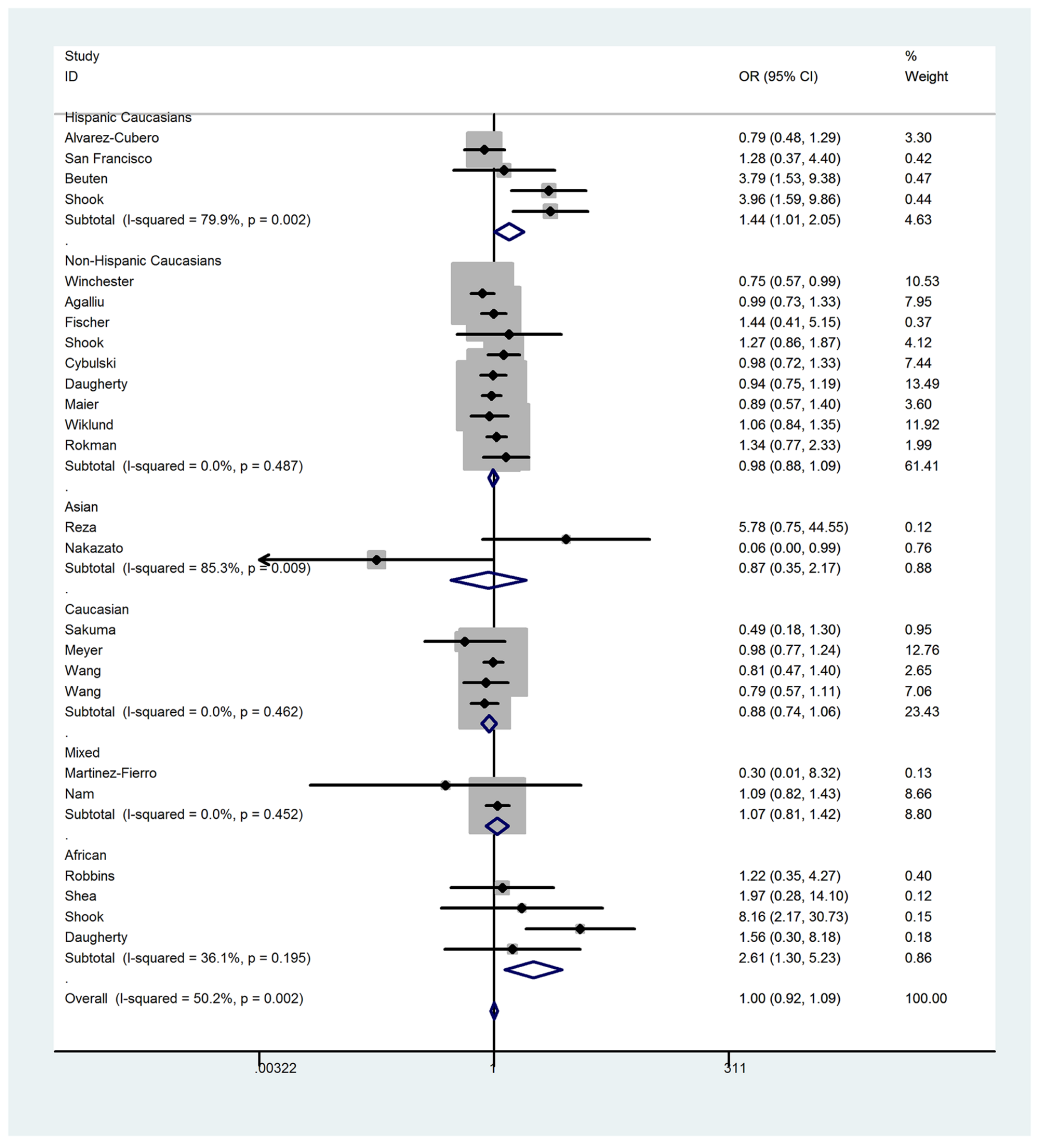
Forest plot of prostate cancer risk associated with RNASEL Arg462Gln polymorphism (recessive genetic model) in the stratified analysis by ethnicity The squares and horizontal lines correspond to the study-specific OR and 95% CI. The area of the squares reflects the weight (inverse of the variance). The diamond represents the summary OR and 95% CI. Separate details were summarized in Table 1.

**Figure 4:**
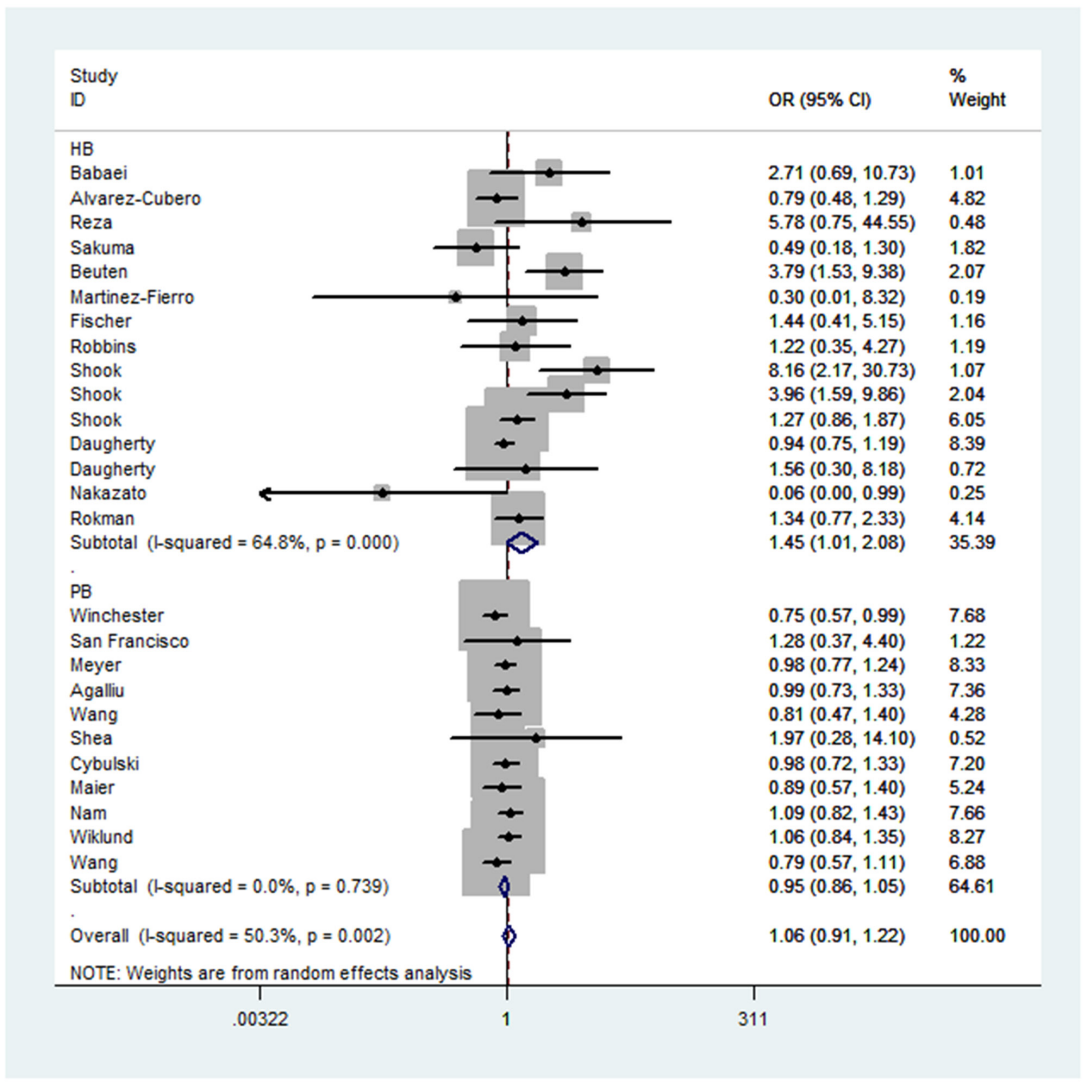
Forest plot of prostate cancer risk associated with RNASEL Arg462Gln polymorphism (recessive genetic model of AA vs. GA + GG) by source of control The squares and horizontal lines correspond to the study-specific OR and 95% CI. The area of the squares reflects the weight (inverse of the variance). The diamond represents the summary OR and 95% CI.

When all the eligible studies were pooled into the analysis (Table 2), no positive association was observed for allelic contrast (fixed-effects OR = 0.99, 95% CI = 0.95 - 1.03, *P*_heterogeneity_ = 0.004, *P* = 0.758, *I*^2^ = 47.9), homozygote comparison (fixed-effects OR = 1.00, 95% CI = 0.91 - 1.09, *P*_heterogeneity_ = 0.001, *P* = 0.968, *I*^2^ = 54.2), heterozygote comparison (fixed-effects OR = 1.01, 95% CI = 0.92 - 1.10, *P*_heterogeneity_ = 0.029, *P* = 0.861, *I*^2^ = 37.6), the dominant genetic model (fixed-effects OR = 0.99, 95% CI = 0.93 - 1.04, *P*_heterogeneity_ = 0.361, *P* = 0.653, *I*^2^ = 7.0), and the recessive genetic model(fixed-effects OR = 1.00, 95% CI = 0.92 – 1.09, *P*_heterogeneity_ = 0.002, *P* = 0.960, *I*^2^ = 50.3). However, in the subgroup analysis by ethnicity, obvious associations between the RNASEL Arg462Gln polymorphism and prostate cancer risk were observed in African descendants for homozygote comparison (fixed-effects OR = 2.59, 95% CI = 1.29 – 5.19, *P*_heterogeneity_ = 0.194, *P* = 0.008, *I*^2^ = 36.3), and the recessive genetic model (fixed-effects OR = 2.61, 95% CI = 1.30 – 5.23, *P*_heterogeneity_ = 0.195, *P* = 0.007, *I*^2^ = 36.1); and for Hispanic Caucasians for the recessive genetic model (fixed-effects OR = 1.44, 95% CI = 1.01 - 2.05, *P*_heterogeneity_ = 0.002, *P* = 0.046, *I*^2^ = 79.9), homozygote comparison (fixed-effects OR = 1.50, 95% CI = 1.02 – 2.20, *P*_heterogeneity_ = 0.001, *P* = 0.039, *I*^2^ = 82.3), and allelic contrast (fixed-effects OR = 1.18, 95% CI = 1.00 - 1.39, *P*_heterogeneity_ = 0.010, *P* = 0.050, *I*^2^ = 73.5). No association was observed in Asian descendants for allelic contrast (fixed-effects OR = 1.30, 95% CI = 0.93 – 1.83, *P*_heterogeneity_ = 0.004, *P* = 0.126, *I*^2^ = 82.2), homozygote comparison (fixed-effects OR = 1.49, 95% CI = 0.70 – 3.17, *P*_heterogeneity_ = 0.013, *P* = 0.303, *I*^2^ = 76.9), and the recessive genetic model (fixed-effects OR = 1.23, 95% CI = 0.57 – 2.63, *P*_heterogeneity_ = 0.019, *P* = 0.600, *I*^2^ = 74.8); non-Hispanic Caucasians for allelic contrast (fixed-effects OR = 0.99, 95% CI = 0.94 – 1.04, *P*_heterogeneity_ = 0.856, *P* = 0.641, *I*^2^ = 0) homozygote comparison (fixed-effects OR = 0.98, 95% CI = 0.87 – 1.10, *P*_heterogeneity_ = 0.631, *P* = 0.701, *I*^2^ = 0), and the recessive genetic model (fixed-effects OR = 0.98, 95% CI = 0.88 – 1.09, *P*_heterogeneity_ = 0.487, *P* = 0.680, *I*^2^ = 0); and mixed descendants for allelic contrast (fixed-effects OR = 1.01, 95% CI = 0.89 – 1.15, *P*_heterogeneity_ = 0.381, *P* = 0.886, *I*^2^ = 0), homozygote comparison (fixed-effects OR = 1.06, 95% CI = 0.79 – 1.42, *P*_heterogeneity_ = 0.453, *P* = 0.692, *I*^2^ = 0), and the recessive genetic model (fixed-effects OR = 1.07, 95% CI = 0.81 – 1.42, *P*_heterogeneity_ = 0.452, *P* = 0.610, *I*^2^ = 0) (Figure 3). Furthermore, a significant association was also observed under the recessive genetic model (random-effects OR = 1.45, 95% CI = 1.01 – 2.08, *P*_heterogeneity_ < 0.001, *P* = 0.046, *I*^2^ = 64.8) between the RNASEL polymorphism and hospital-based controls (Figure 4). Interestingly, in a stratified analysis by the type of prostate cancer, a positive association was observed in familial prostate cancer for allelic contrast (fixed-effects OR = 0.89, 95% CI = 0.79 – 0.99, *P*_heterogeneity_ = 0.209, *P* = 0.028, *I*^2^ = 31.8) and homozygote comparison (fixed-effects OR = 0.77, 95% CI = 0.61 – 0.98, *P*_heterogeneity_ = 0.136, *P* = 0.037, *I*^2^ = 42.9), but not in sporadic prostate cancer for allelic contrast (fixed-effects OR = 1.02, 95% CI = 0.94 – 1.10, *P*_heterogeneity_ = 0.774, *P* = 0.671, *I*^2^ = 0) and homozygote comparison (fixed-effects OR = 1.06, 95% CI = 0.89 – 1.27, *P*_heterogeneity_ = 0.640, *P* = 0.503, *I*^2^ = 0).

**Table 2:** Stratified analyses of the RNASEL Arg462Gln polymorphism on prostate cancer risk

Variables	N^a^	Cases/	A-allele vs. G-allele				AA vs. GG				AA vs. GA+GG			
		Controls	OR(95%CI)	*P*	*P*_heter_^b^	*I*^2^	OR(95%CI)	*P*	*P*_heter_^b^	*I*^2^	OR(95%CI)	*P*	*P*_heter_^b^	*I*^2^
Total	26	11522/10976	0.99(0.95-1.03	0.758	0.004	47.9	1.00(0.91-1.09	0.968	0.001	54.2	1.00(0.92-1.09	0.960	0.002	50.3
Ethnicity														
Asian	3	322/204	1.30(0.93-1.83	0.126	0.004	82.2	1.49(0.70-3.17	0.303	0.013	76.9	1.23(0.57-2.63	0.600	0.019	74.8
African	4	639/1273	1.12(0.91-1.37	0.281	0.056	60.3	2.59(1.29-5.19	0.008	0.194	36.3	2.61(1.30-5.23	0.007	0.195	36.1
Caucasian	4	2511/1996	0.92(0.84-1.00	0.058	0.371	4.4	0.84(0.70-1.02	0.081	0.319	14.6	0.88(0.74-1.06	0.173	0.462	0
Hispanic Caucasians	4	626/725	1.18(1.00-1.35	0.050	0.010	73.5	1.50(1.02-2.20	0.039	0.001	82.3	1.44(1.01-2.05	0.046	0.002	79.9
Non-Hispanic Caucasians	9	6417/5675	0.99(0.94-1.04	0.641	0.856	0	0.98(0.87-1.10	0.701	0.631	0	0.98(0.88-1.09	0.680	0.487	0
Mixed	2	1007/1103	1.01(0.89-1.15	0.886	0.381	0	1.06(0.79-1.42	0.692	0.453	0	1.07(0.81-1.42	0.610	0.452	0
Source of control														
Hospital-based	15	3261/3848	1.06(0.98-1.14	0.120	0.001	63.1	1.47(0.99-2.20	0.059	<0.001^c^	67.9	1.45(1.01-2.08	0.046	<0.001^c^	64.8
Population-based	11	8261/7128	0.97(0.92-1.01	0.169	0.802	0	0.94(0.84-1.04	0.235	0.709	0	0.95(0.86-1.05	0.296	0.739	0
Type of prostate cancer														
Sporadic Pca	6	2838/2934	1.02(0.94-1.10	0.671	0.774	0	1.06(0.89-1.27	0.503	0.640	0	1.07(0.91-1.26	0.441	0.679	0
Familial Pca	5	1313/1967	0.89(0.79-0.99	0.028	0.209	31.8	0.77(0.61-0.98	0.037	0.145	31.8	0.81(0.65-1.02	0.070	0.210	31.7

^a^ Number of comparisons

^b^ *P* value of *Q*-test for heterogeneity test(*P*_heter_).

^c^ Random effects model was performed when *P*_heter_ <0.001; otherwise, fixed effects model was used.

### Publication bias

The Egger’s test and Begg’s funnel plot were carried out to assess the publication bias of the literature. No obvious evidence of publication bias was found (A-allele vs. G-allele, t = 1.72, *P* = 0.098; AA vs. GG, t = 1.77, *P* = 0.089; GA vs. GG, t = 1.75, *P* = 0.094; AA + GA vs. AA, t = 1.23, *P* = 0.231; AA vs. GA + GG, t = 1.77, *P* = 0.090)

## DISCUSSION

Published studies have shown evidence that RNASEL is a constitutively expressed latent endo-ribonuclease that mediates proapoptotic and antiviral activities of the IFN-inducible 2-5A system [7–9]. Mutation carriers in the RNASEL gene have loss of heterozygosity and are deficient in functional RNase L activity [33]. However, previous reports showing association between RNASEL polymorphism and prostate cancer susceptibility are contradictory. The general goal of this pooled analysis is to quantitatively analyze previous studies to understand the true relationship between RNASEL polymorphism and prostate cancer. Here, previous case-control studies with information between the RNASEL polymorphism and different types of Caucasians (Hispanic and Non-Hispanic) were included. As a result, some new findings were observed in our meta-analysis.

Our results indicated that the RNASEL Arg462Gln polymorphism may be associated with increased prostate cancer in African descendants (under homozygote comparison and the recessive genetic model) and Hispanic Caucasians (under allelic contrast, homozygote comparison, and the recessive genetic model), but not in Asian descendants and Non-Hispanic Caucasians. Furthermore, in a stratified analysis by source of control, the RNASEL Arg462Gln variant was found to increase prostate cancer risk in hospital-based studies (under the recessive genetic model). Nevertheless, several caveats limit generalization of these results. First, detailed information, such as age, prognostic parameters, environmental factors, and life-style, were not considered. Second, because different types of prostate cancer influence susceptibility, we tried to assess the effect of this polymorphism to different types of prostate cancer but not all data was compatible. Third, positive findings may be published faster than that those with “negative” results, which may result in a time-lag bias [34]. In addition, more environmental interactions, such as smoking habits, dietary factors, hormone exposure, toxins, and infectious agent, need to be added to the meta-analysis in the future.

Other limitations of the meta-analysis need to be addressed. First, while it is possible that the Arg462Gln polymorphism contributes to cancer, the combined effects of multiple environmental or genetic components predominate in the development of carcinoma, and may mask the effect of the polymorphism [35]. Second, the present analysis was based on unadjusted estimates. A more precise analysis with individual data is needed to evaluate combinatorial effects of the polymorphism [36]. Despite these concerns, the current analysis has some advantages compared with the individual studies. First, a substantial number of cases and controls were pooled from different studies, which significantly enhance the statistical power of this analysis. Second, the quality of case-control studies enrolled in our analysis was satisfactory based on the selection criteria. Third, no obvious publication bias was observed, which indicates that the conclusions were relatively stable and the publication bias might not influence the conclusions of the present meta-analysis.

In conclusion, this meta-analysis showed evidence that the RNASEL Arg462Gln polymorphism may contribute to the risk for developing prostate cancer in African descendants and Hispanic Caucasians, but not for other descendants. Further well-designed and prospective studies, particularly focused on gene-environment interactions, are warranted. These future studies should lead to a comprehensive understanding of the association between the RNASEL Arg462Gln polymorphism and prostate cancer risk.

## MATERIALS AND METHODS

### Search strategy and identification of relevant studies

PubMed database searches were conducted using the following keywords: “ribonuclease L” or “RNASEL”, “prostate cancer”, and “polymorphism” (last search updated on March 01, 2017). References of the relevant articles and retrieved paper were also screened by hand search. Eligible studies had to meet all the following criteria: (a) used an unrelated case–control design; (b) contained information about available genotype frequency; (c) published in English; and (d) included the full-text article.

### Data extraction and quality assessment

Data were collected on the genotype of rs486907 G/A (R462Q) according to prostate cancer. For each publication, the data extraction and methodological quality assessment were conducted by two of the investigators independently to ensure accuracy of the data. Disagreement was resolved by discussion between the two investigators. If they could not reach a consensus, the problem was discussed by all investigators to reach a consensus. The following parameters from each study were recorded: first author’s name, publication date, ethnicity, sources of cancer cases and controls, sample size in cases and controls, and the number of cases and controls with wild-type and variant allele, respectively.

### Statistical analysis

Crude ORs with 95% CIs were utilized to evaluate the strength of association between the RNASEL polymorphism and prostate cancer based on genotype frequencies in cases and controls. Five genetic contrasts were used to assess the association: allelic contrast (A-allele vs. G-allele), homozygote comparison (AA vs. GG), heterozygote comparison (GA vs. GG), the dominant genetic model (AA + GA vs. GG), and the recessive genetic model (AA vs. GA + GG). Subgroup analysis was stratified by ethnicity, source of control (hospital-based and population-based), and smoking exposure. We utilized the random effects model and fixed effects model to calculate the pooled OR. Heterogeneity assumption was evaluated by a chi-square-based *Q* test. *P* value lower than 0.001 for the *Q*-test indicates lack of heterogeneity among studies, hence the pooled OR was utilized by the random effects model (DerSimoniane and Laird method [37] or by the fixed-effects model (the Mantel-Haenszel method [38]). HWE was checked by the Pearson chi-square test for goodness of fit. A *Z*-test was used to evaluate statistical significance of the summary OR, and *P* value of ≤ 0.05 was considered significant. We also utilized the *I*^2^ statistic to test heterogeneity, with *I*^2^ >75%, 25–75%, and < 25% to represent high, moderate, and low degree of inconsistency, respectively [39]. We determined significance of the intercept by t-test suggested by Egger (*P* < 0.01 was considered representative of significant publication bias) [40]. All statistical analyses were performed with STATA version 11.0 (Stata Corporation, College Station, TX), utilizing two-sided *P* values.
